# Clinical and economic impact of incisional negative pressure wound therapy in breast surgery: meta-analysis

**DOI:** 10.1093/bjs/znag056

**Published:** 2026-05-05

**Authors:** Christos Kollatos, Helena Sackey, Eirini Pantiora, Denise Vorburger, Nicola Rocco, Catarina Öhrn, Antonios Valachis, Staffan Eriksson, Andreas Karakatsanis

**Affiliations:** Department of Surgical Sciences, Uppsala University, Uppsala, Sweden; Department of Surgery, Section for Breast Surgery, Uppsala University Hospital, Uppsala, Sweden; Department of Molecular Medicine and Surgery, Karolinska Institutet, Stockholm, Sweden; Department of Breast, Endocrine Tumors and Sarcoma Karolinska Comprehensive Cancer Center, Karolinska University Hospital, Stockholm, Sweden; Department of Surgical Sciences, Uppsala University, Uppsala, Sweden; Department of Surgery, Section for Breast Surgery, Uppsala University Hospital, Uppsala, Sweden; Department of Oncology-Pathology, Karolinska Institutet, Stockholm, Sweden; Breast Unit, Cantonal Hospital Winterthur, Winterthur, Switzerland; Department of Advanced Biomedical Sciences, University of Naples Federico II, Naples, Italy; Department of Biomedical and Clinical Sciences, Linköping University, Linköping, Sweden; Department of Surgery, Linköping University Hospital, Linköping, Sweden; Department of Oncology- Faculty of Medicine and Health, Örebro University, Örebro, Sweden; Department of Surgical Sciences, Uppsala University, Uppsala, Sweden; Centre for Clinical Research Västmanland, Uppsala University, Västerås, Sweden; Section for Breast Surgery- Department of Surgery, Västmanlands County Hospital, Västerås, Sweden; Department of Surgical Sciences, Uppsala University, Uppsala, Sweden; Department of Surgery, Section for Breast Surgery, Uppsala University Hospital, Uppsala, Sweden

## Abstract

**Background:**

Wound complications after breast surgery are common and contribute to morbidity and healthcare costs. The economic value of prophylactic incisional negative-pressure wound therapy (iNPWT) remains uncertain. This study evaluated the effectiveness and cost-effectiveness of iNPWT in preventing postoperative wound complications (freedom from complication) following breast surgery through a systematic review and meta-analysis.

**Methods:**

MEDLINE, EMBASE, and the Cochrane Central Register of Controlled Trials were searched without any restrictions up to 5 March 2025. Randomized trials and prospective or retrospective cohort studies comparing prophylactic iNPWT with standard dressings were included. Two reviewers independently extracted data, assessed risk of bias (RoB 2, ROBINS-I), and graded evidence certainty (GRADE). Random-effects meta-analyses were performed, and cost-effectiveness modelling focused on surgical site infection (SSI).

**Results:**

Twenty-nine studies including 4904 patients (1817 iNPWT; 3087 controls) were analysed. iNPWT was associated with higher rates of wound dehiscence prevention (RR 1.07, 95% c.i. 1.03 to 1.12), SSI prevention (RR 1.05, 95% c.i. 1.01 to 1.09), and skin necrosis prevention (RR 1.07, 95% c.i. 1.01 to 1.15). No significant differences were observed for seroma, nipple–areolar complex necrosis, or haematoma. Across outcomes, heterogeneity was substantial and 95% prediction intervals crossed the null, indicating uncertainty in the direction of effect in future settings. Evidence certainty ranged from low to moderate. Cost modelling suggested that iNPWT may be cost-saving only in settings with high SSI-related costs.

**Conclusions:**

Current evidence does not establish consistent clinical benefit of prophylactic iNPWT in breast surgery. Cost-effectiveness appears limited to high-risk or high-cost contexts, supporting selective use.

## Teaser

Prophylactic iNPWT may confer modest reductions in selected wound complications after breast surgery, but benefits are inconsistent. Cost-effectiveness appears limited to high-risk or high-cost contexts, supporting selective rather than routine use.

## Introduction

Breast cancer is the most common cancer among women worldwide, and surgery remains a cornerstone of treatment. Advances in oncoplastic and reconstructive techniques have expanded surgical options, yet postoperative wound complications, including seroma, surgical site infection (SSI), haematoma, wound dehiscence, flap necrosis, and nerve damage, remain frequent^1–3^. These complications carry clinical significance due to associated morbidity, negative cosmetic and psychological outcomes, and the potential delay of adjuvant therapy, particularly in patients with triple-negative breast cancer, in whom delays are linked with worse survival^4–6^, while additionally imposing considerable healthcare costs^7^.

Incisional negative-pressure wound therapy (iNPWT) on primary closed incisions has been proposed as a method to reduce wound complications^8^. Initially developed for open or chronic wounds, iNPWT has more recently been used on closed surgical incisions^9^. Its proposed benefits include protection of the incision, fluid evacuation, reduction of contamination and infection risk, decreased oedema, stimulation of angiogenesis, improved lymphatic and vascular flow, reduced wound-edge tension, improved skin elasticity, and maintenance of a moist healing environment^10–13^.

A prior meta-analysis suggested that iNPWT might reduce SSI across different types of surgery^8^. Regarding breast surgery, the findings were, however, driven by a single study of 50 patients per arm, whereas other breast-specific trials showed no clear benefit^14,15^. Substantial heterogeneity across studies, particularly regarding patient selection, further limits interpretation^1,16^.

The aim of this systematic review and meta-analysis was to provide a comprehensive evaluation of clinical outcomes and cost-effectiveness of iNPWT in breast surgery.

## Methods

### Selection criteria and search strategy

A systematic review and meta-analysis were conducted following the PRISMA statement^17^. The protocol was registered in PROSPERO (ID: CRD420250654302). Search criteria were defined using the PICOS framework^18^:

- P (Population): Patients (women or men) with closed incisions following breast surgery
- I (Intervention/Exposure): Prophylactic iNPWT
- C (Comparison): Standard wound care dressings
- O (Outcome): Prevention (freedom from complication) of wound dehiscence, SSI, seroma, skin necrosis, nipple–areolar complex (NAC) necrosis, or haematoma
- S (Study design): RCTs, prospective or retrospective cohort studies

Studies were included if they reported at least one predefined outcome. Exclusion criteria were NPWT for open or infected wounds, non-breast surgery, animal studies, procedures outside the operating theatre, review articles, commentaries, and studies lacking measures of association. No restrictions were placed on publication year, language, or sample size.

A librarian at the Uppsala University Library, Sweden, conducted the literature search, which included Medline (PubMed), Excerpta Medica Database (EMBASE), and the Cochrane Central Register of Controlled Trials (CENTRAL). All searches were completed on 5 March 2025 and full search strategies are detailed in *Appendix S1* (*Supplementary material*). Duplicates were removed in Rayyan^19^. Two reviewers (C.K. and H.S.) independently screened titles/abstracts and assessed full texts; disagreements were resolved by consensus or by the senior author (A.K.).

### Data extraction and analysis approach

Extracted study characteristics included authorship, publication year, design, centre type, surgical indication/procedure, and clinical outcomes. Prevention of wound dehiscence was the primary outcome; secondary outcomes were prevention of SSI, seroma, skin necrosis, NAC necrosis, and haematoma. Definitions followed each study’s reporting.

Two reviewers extracted data using a standardized form and cross-checked accuracy. Intervention details included device type, pressure settings, duration, follow-up period, and industry involvement. When unspecified, default pressures per manufacturer were assumed: −80 mmHg for PICO (Smith & Nephew, Hull, UK) and Avelle (Convatec, London, UK), and −125 mmHg for PREVENA (KCI, San Antonio, TX, USA).

Risk ratios (RRs) and pooled absolute risk differences (ARD) with 95% confidence intervals were calculated using random-effects models (Hartung–Knapp–Sidik–Jonkman)^20^. This model was appropriate given the expected variability in effect sizes due to differences in study design, populations, and contexts^21^. Because the outcome was defined as prevention of the complication, an RR greater than 1 indicates higher success rates in the intervention group. A 95% prediction interval was also calculated to estimate potential effect sizes in future studies^22^, capturing both effect uncertainty and interstudy variation. Additionally, the risk difference was reported as an absolute measure of effect^23^. Pooled proportions with respective 95% confidence intervals were calculated with the metaprop package^24^.

Heterogeneity was assessed using the *I*^2^ statistic and tau-squared (*T*^2^) was used to quantify between-study variance^25^. Subgroup analyses were conducted separately for RCTs and observational studies.

To explore sources of heterogeneity, a mixed-effects meta-regression was planned using several study-level covariates. The main covariates were age, BMI, and the difference in smoking prevalence between iNPWT and control groups. Secondary covariates included surgical indication, type of operation, and device type. Inclusion of each secondary variable in the final model was contingent on statistical feasibility, specifically sufficient between-study variability and model convergence. Model stability was assessed using pseudo-*R*^2^  ^26^. A leave-one-out sensitivity analysis tested the robustness of the pooled effect by repeatedly removing each individual study from the data set and re calculating the overall effect.

Risk of bias was assessed using the Cochrane Risk of Bias-2 (RoB 2) tool^27^ for RCTs and the ROBINS-I tool for non-randomized studies^28^. Potential publication bias was evaluated using a comparison-adjusted funnel plot and Egger’s regression test. The Grading of Recommendations, Assessment, Development and Evaluations (GRADE) approach was used to evaluate evidence certainty across five domains: risk of bias, imprecision, inconsistency, indirectness, and publication bias^29^. Two reviewers (D.V. and C.K.) independently applied the above methods to assess risk of bias and evaluate the certainty of evidence, with any disagreements resolved through discussion and the senior author (A.K.) providing the final decision when necessary.

A cost-effectiveness analysis focusing exclusively on SSI was conducted to evaluate the economic impact of iNPWT compared with standard wound dressings in breast surgery, using a decision tree model^30^. The parameters were based on institutional audits in the authors’ institutes and SSI was selected as the outcome because relevant cost data for the device and complications respectively were available in prior literature^7,31^.

The model compared two strategies: prophylactic iNPWT and standard wound dressings. Pooled ARD estimates from the meta-analysis were used to calculate the number needed to treat (NNT) to prevent one SSI. Cost data included fixed costs of iNPWT (device and nursing care) and standard dressings, as well as surgical costs with and without SSI complications. Incremental cost per patient and net cost per SSI prevented were calculated by combining incremental treatment costs with cost savings from preventing an SSI. Sensitivity analyses assessed variations in ARD and SSI-related costs to evaluate the robustness of economic outcomes. All costs are reported in Euro (€), using conversion rates current as of May 2025.

All statistical analyses were performed using Stata Statistical Software, Release 17 (StataCorp LLC, College Station, TX, USA).

## Results

### Study selection

**Fig. 1:**
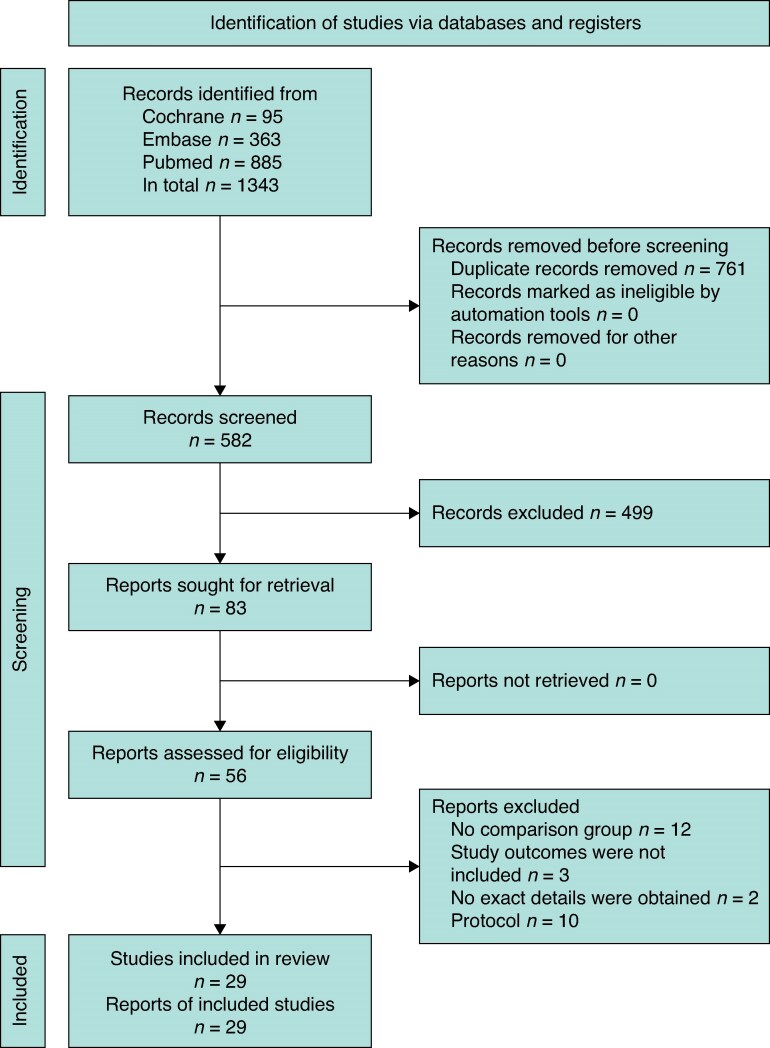
PRISMA flow diagram

The flowchart for the systematic review and meta-analysis is presented in *Fig. 1*. Of 1343 publications, 29 articles^15,32–59^ (2.2%) were included in the final analysis. All authors reached consensus on study inclusion. Collectively, the studies reported data from 4904 patients: 1817 (37.1%) in the iNPWT group and 3087 (62.9%) in the non-iNPWT group. A total of 1817 breasts (38.5%) were included in the iNPWT group and 2906 (61.5%) in the non-iNPWT group.

The included studies consisted of five RCTs^15,37,44,50,55^ (17.2%), eight prospective non-randomized studies^34,38,45,46,49,51,53,59^ (27.6%), 13 retrospective studies^32,33,35,39,40,43,47,48,52,54,56–58^ (44.8%), and one retrospective study with propensity score matching^36^ (3.4%) (*Table 1*). Of the 29 studies, 26^15,32–43,45–54,56,58,59^ (89.6%) were single centre. Fourteen reported on mastectomy^15,32,33,35,36,38,40,43,46,48,50,54–56^ (48.3%) and four on reduction mammaplasty^37,44,47,49^ (13.8%). One study^34^ (3.4%) included both procedures, whereas two others^45,52^ (6.9%) involved patients undergoing contralateral reduction mammaplasty or mastopexy during oncoplastic breast reduction. Two studies^39,59^ (6.9%) focused on oncological surgery in patients with risk factors and four^41,42,51,53^ (13.8%) did not report the type of surgery. The surgical indication was breast cancer in 14 studies^34,36–38,40,41,45,50–52,56–59^ (48.3%), gender-affirming mastectomy in two^15,32^ (6.9%), reduction mammoplasty in two^44,47^ (6.9%) and any categories in two studies^33,55^ (6.9%). Nine studies^35,39,42,43,46,48,49,53,54^ (31.0%) did not report the surgical indication.

**Table 1:** Characteristics of included studies: design, institution type, surgical procedure, and surgical indication

Study	Institution, study design	Surgery type, indication
Abu El Hawa *et al.*^32^	Single centre, retrospective	Gender-affirming mastectomies
Akhter *et al.*^33^	Single centre, retrospective	Immediate breast reconstruction for any categories
Al-Ishaq *et al.*^34^	Single centre, prospective	Mastectomy/reduction mammaplasty for breast cancer
Alameddine *et al.*^35^	Single centre, retrospective	Immediate breast reconstruction after radiotherapy (indication was not reported)
Alameddine *et al.*^36^	Single centre, retrospective with propensity score matching	Immediate breast reconstruction for breast cancer
Casella *et al.*^37^	Single centre, RCT	Wise-pattern reduction mammaplasty for breast cancer
De Rooij *et al.*^38^	Single centre, prospective	Mastectomy for breast cancer
Diaz *et al.*^39^	Single centre, retrospective	Oncological breast surgery with risk factors (indication was not reported)
Esen *et al.*^40^	Single centre, retrospective	Mastectomy for breast cancer
Fernandes *et al.*^41^	Single centre; study design was not reported	Surgery type was not reported. Indication was breast cancer
Ferrando *et al.*^59^	Single centre, prospective	Oncological breast surgery with risk factors for breast cancer
Fogacci *et al.*^42^	Single centre, study design was not reported	Not reported
Gabriel *et al.*^43^	Single centre, retrospective	Immediate breast reconstruction (indication was not reported)
Galiano *et al.*^44^	Multicentre, RCT	Reduction mammaplasty for functional benefit
Holt *et al.*^45^	Single centre, prospective	Oncological breast surgery for breast cancer
Irwin *et al.*^46^	Single centre, prospective	Immediate breast reconstruction (indication was not reported)
Johnson *et al.*^47^	Single centre, retrospective	Reduction mammaplasty for functional benefit
Kim *et al.*^48^	Single centre, retrospective	Mastectomy (indication was not reported)
Lane *et al.*^49^	Single centre, prospective	Reduction mammaplasty (indication was not reported)
Lauritzen *et al.*^50^	Single centre, RCT	Immediate breast reconstruction for breast cancer
Nip *et al.*^51^	Single centre, prospective	Type of surgery not reported. Indication was breast cancer
Ockerman *et al.*^52^	Single centre, retrospective	Reduction mammaplasty/mastopexy post lumpectomy for breast cancer
Pellino *et al.*^53^	Single centre, prospective	Not reported
Pieri *et al.*^54^	Single centre, retrospective	Mastectomy (indication was not reported)
Pieszko *et al.*^55^	Multicentre, RCT	Immediate breast reconstruction for any categories
Ryu *et al.*^56^	Single centre, retrospective	Immediate breast reconstruction for breast cancer
Timmermans *et al.*^15^	Single centre, RCT	Gender-affirming mastectomies
Tormey *et al.*^57^	Multicentre, retrospective	Oncological breast surgery for breast cancer
Wareham *et al.*^58^	Single centre, retrospective	Oncological breast surgery for breast cancer

Device usage varied: PREVENA was used in 11 studies^32,33,36,40,43,47,50,52,54,58,59^ (37.9%), PICO in nine^15,34,39,42,44–46,53,56^ (31.0%), and Avelle in two^38,55^ (6.9%). In seven studies (24.1%), the iNPWT device was not specified^35,37,41,48,49,51,57^. Industry funding was reported in six studies^38,43,44,52,55,58^ (20.7%), explicitly absent in 11^15,32,33,36,37,40,47,50,51,53^ (37.9%), and not reported in 12^34,35,39,41,42,45,46,48,49,54,56,57^ (41.4%) (*Table S1*).

The median age in the iNPWT group was 45 years (i.q.r. 23–52.4), compared with 49.8 years (i.q.r. 42.2–55.8) in the non-iNPWT group. Median BMI was 27 (i.q.r. 25.5–28.2) for the iNPWT group and 27.5 (i.q.r. 24.8–28.2) for the non-iNPWT group. Smoking was reported in 88 of 1817 patients (4.84%) in the iNPWT group and 145 of 3087 patients (4.70%) in the non-iNPWT group.

### Prevention of wound dehiscence

**Fig. 2:**
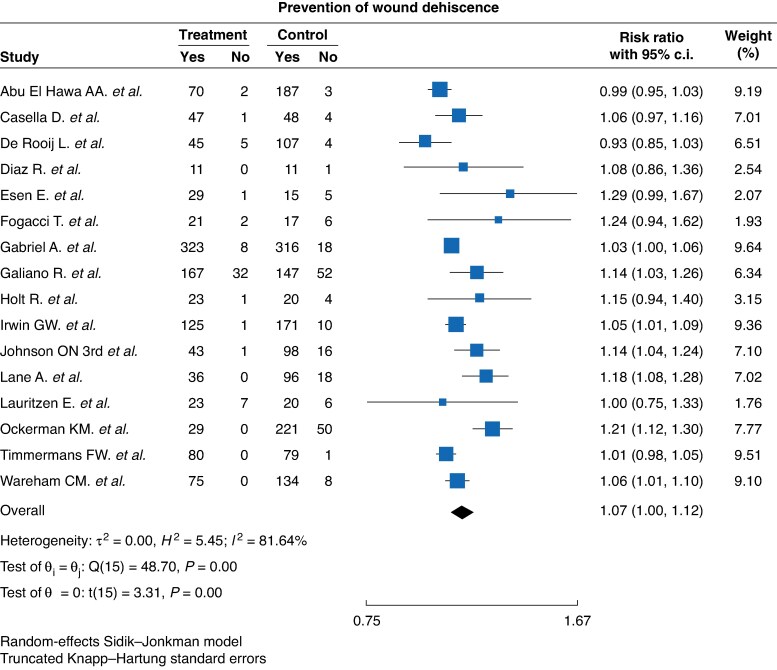
Forest plot illustrating the effect of incisional negative pressure wound therapy (iNPWT) on prevention of wound dehiscence

Sixteen studies including 3101 patients (1208 iNPWT; 1893 controls) reported wound dehiscence. The pooled prevention incidence was 97% (95% c.i. 94% to 99%) with iNPWT *versus* 90% (95% c.i. 85% to 94%) in controls. Meta-analysis showed iNPWT significantly prevented dehiscence compared with standard care (RR = 1.074, 95% c.i. 1.029 to 1.120, *P* < 0.001) (*Fig. 2*). Heterogeneity was substantial (*I*^2^ = 81.64%, τ^2^ = 0.004), with a 95% prediction interval of 0.923–1.249. Leave-one-out analysis confirmed stable estimates (RR 1.062–1.082).

The pooled absolute risk difference was 0.070 (95% c.i. 0.031 to 0.109, *P* = 0.001). In subgroup analyses, RCTs showed RR 1.049 (95% c.i. 0.965 to 1.141; *I*^2^ = 41.2%), whereas observational studies showed RR 1.082 (95% c.i. 1.023 to 1.144; *I*^2^ = 84.5%). The funnel plot suggested slight asymmetry, but Egger’s test was not significant (β_1_ = 0.98, *P* = 0.112). Meta-regression did not identify age, BMI, or smoking as moderators (*Table S2*). Residual heterogeneity remained substantial (*I*^2^ = 67.32%), with minimal explanatory power (*R*^2^ = 0.00%).

### Prevention of surgical site infection

**Fig. 3:**
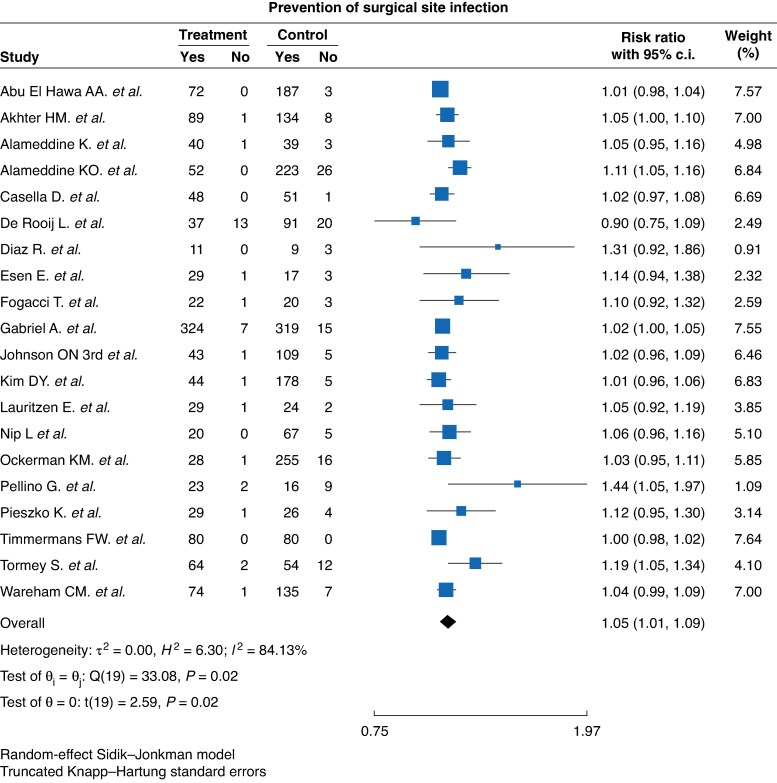
Forest plot illustrating the effect of incisional negative pressure wound therapy (iNPWT) on prevention of surgical site infection

Twenty studies (1192 iNPWT; 2184 controls) evaluated SSI. The pooled prevention incidence was 98% (95% c.i. 96% to 99%) with iNPWT *versus* 93% (95% c.i. 90% to 95%) in controls. Random-effects analysis showed significantly improved SSI prevention with iNPWT (RR = 1.048, 95% c.i. 1.013 to 1.089, *P* = 0.02) (*Fig. 3*). Heterogeneity was high (*I*^2^ = 84.13%), with a 95% prediction interval of 0.913–1.203. Leave-one-out analysis confirmed stable effects (RR 1.041–1.051).

The absolute risk difference was 0.048 (95% c.i. 0.020 to 0.076, *P* = 0.003). Subgroup analyses showed no significant effect in RCTs (RR 1.018, 95% c.i. 0.966 to 1.072; *I*^2^ = 41.4%) but a significant effect in observational studies (RR 1.054, 95% c.i. 1.015 to 1.095; *I*^2^ = 85.5%). The funnel plot appeared symmetrical (*Fig. S2*), although Egger’s test indicated small-study effects (β_1_ = 1.21, *P* = 0.006). Meta-regression identified no significant predictors (*Table S2*); residual heterogeneity decreased (*I*^2^ = 38.38%; *R*^2^ = 36.92%).

### Prevention of seroma

Twenty studies (1334 iNPWT; 2247 controls) evaluated seroma. The pooled incidence was 92% (95% c.i. 85% to 96%) with iNPWT *versus* 85% (95% c.i. 78% to 90%) in controls. Random-effects modelling showed a non-significant RR of 1.082 (95% c.i. 0.993 to 1.179, *P* = 0.08) (*Fig. S3*). Heterogeneity was substantial (*I*^2^ = 96.68%), with a prediction interval of 0.763–1.533. Leave-one-out analysis confirmed no undue influence (RR 1.067–1.090).

Absolute risk difference was 0.064 (95% c.i. 0.009 to 0.119, *P* < 0.001). In subgroup analyses, RCTs showed RR 1.037 (95% c.i. 0.742 to 1.449; *I*^2^ = 93.5%), and observational studies showed RR 1.085 (95% c.i. 1.010 to 1.165; *I*^2^ = 92.9%). Funnel plot (*Fig. S4*) showed asymmetry; Egger’s test suggested small-study effects (β_1_ = 1.56, *P* = 0.003). Multivariable meta-regression identified an association for the Avelle device (*P* = 0.022), whereas other covariates were not significant (*Table S2*).

### Prevention of skin necrosis

Eleven studies (918 iNPWT, 1400 controls) evaluated skin necrosis. The pooled incidence was 95% (95% c.i. 91% to 98%) with iNPWT *versus* 88% (95% c.i. 81% to 94%) in controls. Random-effects modelling showed RR = 1.073 (95% c.i. 1.005 to 1.147, *P* = 0.001) (*Fig. S5*). Heterogeneity was high (*I*^2^ = 87.81%). Leave-one-out analysis ranged 1.054–1.080. Absolute risk difference was 0.064 (95% c.i. 0.009 to 0.210, *P* < 0.001). In subgroup analyses, RCTs showed RR 1.03 (95% c.i. 0.983 to 1.079; *I*^2^ = 6.3%), and observational studies showed RR 1.086 (95% c.i. 0.985 to 1.198; *I*^2^ = 89.9%). Funnel plot was asymmetrical towards the right (*Fig. S6*); Egger’s test was non-significant (β_1_ = 0.99, *P* = 0.25). Younger age (β = −0.188, *P* = 0.039) and lower BMI (β = −0.169, *P* = 0.042) increased effectiveness; smoking had no effect (*Table S2*). Residual heterogeneity was high (*I*^2^ = 64.88%, *R*^2^ = 20.81%).

### Prevention of NAC necrosis

Five studies (310 iNPWT, 859 controls) evaluated NAC necrosis; no RCTs were available. The pooled incidence was 97% (95% c.i. 91% to 100%) with iNPWT *versus* 95% (95% c.i. 84% to 100%) in controls. Random-effects modelling showed no significant benefit (RR = 1.027, 95% c.i. 0.930 to 1.135, *P* = 0.499) (*Fig. S7*). Heterogeneity was high (*I*^2^ = 91.09%), prediction interval 0.786–1.342. Leave-one-out analysis ranged 1.000–1.042. Absolute risk difference was 0.024 (95% c.i. −0.051 to 0.100, *P* = 0.527). Funnel plot showed asymmetry towards the left (*Fig. S8*); Egger’s test was non-significant (β_1_ = 3.25, *P* = 0.089). Meta-regression identified no significant predictors; residual heterogeneity *I*^2^ = 11%, *R*^2^ = 97.5%.

### Prevention of haematoma

Fifteen studies (1215 iNPWT, 1871 controls) evaluated haematoma. The pooled incidence was 98% (95% c.i. 96% to 100%) with iNPWT *versus* 96% (95% c.i. 93% to 98%) in controls. Random-effects modelling showed RR = 1.017 (95% c.i. 1.002 to 1.032, *P* = 0.545), *I*^2^ = 59.47%, prediction interval 0.959–1.077 (*Fig. S9*). Leave-one-out analysis RR 1.062–1.082. Absolute risk difference was 0.018 (95% c.i. 0.005 to 0.032, *P* = 0.311). In subgroup analyses, RCTs showed RR 1.008 (95% c.i. 0.994 to 1.022; *I*^2^ = 3.8%), and observational studies showed RR 1.021 (95% c.i. 0.996 to 1.046; *I*^2^ = 72.1%). Funnel plot (*Fig. S10*) was asymmetrical towards the left; Egger’s test indicated small-study effects (β_1_ = 0.99, *P* = 0.009). Meta-regression found no predictors; residual heterogeneity *I*^2^ = 20.93%, *R*^2^ = 0%).

Key results are summarized in *Table 2*.

**Table 2:** Meta-analysis results: summary of key outcomes

Outcome	*N*	*n*_i_	*n*_c_	RR (95% c.i.)	*P* (RR)	Prediction interval	ARD, (95% c.i.)	*P* (risk diff)	Overall certainty of evidence*
Wound dehiscence	16	1208	1893	1.074 (1.029–1.120)	<0.001	0.923–1.249	7.0% (3.1%–10.9%)	0.001	⬤⬤⬤◯ Moderate
Surgical site infection	20	1192	2184	1.048 (1.013–1.089)	0.020	0.913–1.203	4.8% (2.0%–7.6%)	0.003	⬤⬤◯◯ Low
Seroma	20	1334	2247	1.082 (0.993–1.179)	0.080	0.763–1.533	6.4% (0.9%–11.9%)	<0.001	⬤◯◯◯ Very low
Skin necrosis	11	918	1400	1.073 (1.005–1.147)	0.001	0.868–1.327	6.4% (0.9%–21.0%)	<0.001	⬤⬤◯◯ Low
NAC necrosis	5	310	859	1.027 (0.930–1.135)	0.499	0.786–1.342	2.4% (−5.1%–10.0%)	0.527	⬤◯◯◯ Very low
Haematoma	15	1215	1871	1.017 (1.002–1.032)	0.545	0.959–1.077	1.8% (0.5%–3.2%)	0.311	⬤⬤◯◯ Low

*n*_i_, Number of patients in the intervention group (incisional negative pressure wound therapy); *n*_c_, Number of patients in the control group (standard wound care dressings); RR, risk ratio; ARD, absolute risk difference. *Certainty of evidence assessed using the GRADE approach. More detailed information for each outcome is provided in *Table S5*.

### Review of risk of bias

RCTs were mostly low risk or some concerns, especially in randomization, deviations, and outcome measurement. One RCT had serious risk due to outcome measurement bias^50^ (*Table S3*).

Non-randomized studies more frequently had moderate-to-serious bias, particularly in confounding and participant selection, with some measurement and reporting bias; only 8/25 studies were moderate risk (*Table S4*). GRADE ratings were downgraded for design limitations, particularly for outcomes based mainly on non-randomized studies (SSI, seroma, skin necrosis, NAC necrosis, haematoma) (*Table S5*).

### Cost-effectiveness analysis focused on surgical site infection

The absolute risk difference of 0.048 corresponded to an NNT of 21 patients to prevent one SSI. The fixed cost of iNPWT per patient was estimated at €220, comprising the device cost (€166) and nursing care for dressing changes (€54). In comparison, standard care dressings cost €9 per patient^31^. Therefore, the incremental cost of iNPWT per patient compared to standard dressings was €211.

The median total cost for patients with an SSI was $16 882, compared with $6123 for those without infection. The difference ($10 759) represents the incremental cost attributable to SSI, corresponding to approximately €10 008 after currency conversion. To reflect real-world variability, two SSI-related cost scenarios were modelled: a low-cost scenario (€3481) based on conservative estimates, and a median-cost scenario (€10 005) based on the calculated difference between patients with and without SSI.

Sensitivity analyses under four scenarios, combining low and median values of ARD and SSI-associated costs, demonstrated that the net cost of iNPWT per SSI prevented ranged from a cost saving of €5574 (median ARD and median SSI cost) to a net cost of €7069 (low ARD and low SSI cost) (*Table S6*).

## Discussion

This meta-analysis found that the prophylactic use of iNPWT in breast surgery showed modest benefit in preventing wound dehiscence, surgical site infection, and skin necrosis. However, these findings need to be interpreted in the context of significant limitations, including substantial heterogeneity, residual variability, and prediction intervals that do not support high certainty in a positive effect. Most studies reporting benefit are retrospective and compare groups without control for baseline characteristics, underscoring the need for well-conducted RCTs to clarify whether a causative effect exists and determine its magnitude.

According to the GRADE framework, outcomes supported mainly by non-randomized studies that is surgical site infection, seroma, skin necrosis, NAC necrosis, and haematoma, were rated ‘low to very low certainty’, whereas wound dehiscence reached ‘moderate certainty’, despite the availability of randomized trial data. Although these findings indicate a potential benefit of iNPWT, the overall strength of evidence remains limited due to prediction intervals crossing the null, indicating that the true effect may vary substantially across settings. Therefore, even these outcomes should be interpreted with caution, and the overall certainty of evidence remains limited.

Cost-effectiveness analysis further contextualized these results. Preventing surgical site infection has substantial clinical and economic impact. Under high-cost assumptions (€10 005 per infection), iNPWT was cost-saving (€5574 per infection prevented), whereas under lower-cost assumptions (€3481 per infection), it resulted in a net cost of €7069 per infection prevented. Cost-effectiveness is thus closely linked to baseline infection risk and healthcare cost structures. Therefore, although iNPWT may benefit high-risk patients or high-cost settings, the present results suggest that routine use in breast surgery is not justified. On the other hand, even modest reductions in complications may be clinically meaningful for patients where wound healing affects timely adjuvant therapy; however, this is yet to be assessed in a clinical trial, therefore, no direct such claim can be made presently.

This analysis is the largest and most detailed synthesis of iNPWT in breast surgery, incorporating 29 studies and nearly 5000 patients. Although leave-one-out sensitivity analysis did not suggest a significant change in outcomes, all prediction intervals included the null, suggesting benefits are unlikely to be consistent across populations. Further high-quality randomized trials targeting well-defined high-risk groups are needed.

Limitations include underreporting of co-morbidities, which restricted subgroup analyses, and substantial clinical and methodological heterogeneity across studies. The predominance of non-randomized and retrospective designs introduces selection bias, confounding by indication, and incomplete adjustment for baseline risk, meaning that observed associations should not be interpreted as causal. Differences in outcome definitions, follow-up periods, perioperative antibiotic protocols, drain use, and NPWT device parameters further contributed to heterogeneity and may have introduced misclassification bias, reducing comparability across studies. Because multiple outcomes were examined and exploratory meta-regressions were performed across several covariates without formal adjustment for multiplicity, the statistically significant moderator findings should be interpreted cautiously. Additionally, the cost-effectiveness analysis relied on U.S. SSI-related cost estimates from 2008, which may not reflect contemporary or international healthcare settings. No inflation adjustment was performed because comparable contemporary cost data were not available. Although two cost scenarios were modelled to capture variability, differences in device pricing, reimbursement structures, and healthcare resource utilization may limit the generalizability of the economic findings.

Current evidence is insufficient to determine whether iNPWT provides consistent or clinically meaningful benefit in breast surgery. Although modest improvements in selected outcomes are possible, substantial heterogeneity, low certainty of evidence, and prediction intervals crossing the null limit confidence in these findings. High-quality randomized trials are needed before firm recommendations can be made.

## Supplementary Material

znag056_Supplementary_Data

## Data Availability

The data supporting the findings of this study are available from the corresponding author upon reasonable request.
