# Supplementary material for: Clinical and economic impact of incisional negative pressure wound therapy in breast surgery: meta-analysis
Source: Br J Surg. 2026 May 5;113(6):znag056. doi: 10.1093/bjs/znag056 (PMC13244580; doi:10.1093/bjs/znag056)
Supplement: znag056_Supplementary_Data [file znag056_supplementary_data.zip › Supplementary_material.docx]

**Clinical and Economic Impact of Incisional Negative Pressure Wound Therapy in Breast Surgery: Meta-analysis**

Christos Kollatos^1,2^, Helena Sackey^3,4^, Eirini Pantiora^1,2,5^, Denise Vorburger^6^, Nicola Rocco^7^, Catarina Öhrn^8,9^, Antonios Valachis^10^, Staffan Eriksson^1,11,12^, Andreas Karakatsanis^1,2^

^1^ Department of Surgical Sciences, Uppsala University, Uppsala, Sweden.

^2^ Department of Surgery, Section for Breast Surgery, Uppsala University Hospital, Uppsala, Sweden.

^3^ Department of Molecular Medicine and Surgery, Karolinska Institutet, Stockholm, Sweden.

^4^ Department of Breast, Endocrine Tumors and Sarcoma Karolinska Comprehensive Cancer Center, Karolinska University Hospital, Stockholm, Sweden.

^5^ Department of Oncology-Pathology, Karolinska Institutet, Stockholm, Sweden

^6^ Breast Unit, Cantonal Hospital Winterthur, Winterthur, Switzerland.

^7^ Department of Advanced Biomedical Sciences, University of Naples Federico II, Naples, Italy

^8^ Department of Biomedical and Clinical Sciences, Linköping University, Linköping, Sweden.

^9^ Department of Surgery, Linköping University Hospital, Linköping, Sweden.

^10^ Department of Oncology- Faculty of Medicine and Health, Örebro University, Örebro, Sweden.

^11^ Centre for Clinical Research Västmanland, Uppsala University, Västerås, Sweden

^12^ Section for Breast Surgery- Department of Surgery, Västmanlands County Hospital, Västerås, Sweden.

**Corresponding Author:** Christos Kollatos, Department of Surgical Sciences, Uppsala University, Akademiska Sjukhusvägen, Ingång 70, Uppsala 751 85, Sweden, email: [christos.kollatos@uu.se](mailto:christos.kollatos@uu.se). **ORCID ID** 0000-0001-5601-1794

**Supplementary Materials - Index**

| **Supplementary Appendixes** |  |
| --- | --- |
| Appendix 1. Search strategies | *page 2-4* |
| **Supplementary Figures and Tables** |  |
| Supplementary Figures 1-10 | *page 5-14* |
| Supplementary Tables 1-6 | *page 15-23* |
| **References** | *page 24-28* |

**Appendix 1. Search strategies**

**Pubmed**

*Search nr 1 (Results: 593)*

(((((negative[All Fields] AND (‘‘pressure’’[MeSH Terms] OR ‘‘pressure’’[All Fields])) OR (‘‘negative-pressure wound therapy’’[MeSH Terms] OR (‘‘negative-pressure’’[All Fields] AND ‘‘wound’’[All Fields] AND ‘‘therapy’’[All Fields]) OR ‘‘negative-pressure wound

therapy’’[All Fields] OR (‘‘negative’’[All Fields] AND ‘‘pressure’’[All Fields] AND ‘‘dressing’’[All Fields]) OR ‘‘negative pressure dressing’’[All Fields])) OR VAC[All

Fields]) OR PICO[All Fields]) OR (‘‘negative-pressure wound therapy’’[MeSH Terms] OR (‘‘negative-pressure’’[All Fields] AND ‘‘wound’’[All Fields] AND ‘‘therapy’’[All Fields]) OR ‘‘negative-pressure wound therapy’’[All Fields] OR (‘‘negative’’[All Fields] AND

‘‘pressure’’[All Fields] AND ‘‘wound’’[All Fields] AND ‘‘therapy’’[All Fields]) OR ‘‘negative pressure wound therapy’’[All Fields])) AND ((((((‘‘breast’’[MeSH Terms]

OR ‘‘breast’’[All Fields]) AND (‘‘surgery’’[Subheading] OR ‘‘surgery’’[All Fields] OR ‘‘surgical procedures, operative’’[MeSH Terms] OR (‘‘surgical’’[All Fields] AND

‘‘procedures’’[All Fields] AND ‘‘operative’’[All Fields]) OR ‘‘operative surgical procedures’’[All Fields] OR ‘‘surgery’’[All Fields] OR ‘‘general surgery’’[MeSH Terms] OR (‘‘general’’[All Fields] AND ‘‘surgery’’[All Fields]) OR ‘‘general surgery’’[All Fields])) OR (‘‘mammaplasty’’[MeSH Terms] OR ‘‘mammaplasty’’[All Fields] OR (‘‘breast’’[All Fields] AND ‘‘reconstruction’’[All Fields]) OR ‘‘breast reconstruction’’[All Fields])) OR ((‘‘breast’’[MeSH Terms] OR ‘‘breast’’[All Fields]) AND augmentation[All Fields])) OR ((‘‘breast’’[MeSH Terms] OR ‘‘breast’’[All Fields]) AND reduction[All Fields])) OR

(‘‘mastectomy, simple’’[MeSH Terms] OR (‘‘mastectomy’’[All Fields] AND ‘‘simple’’[All Fields]) OR ‘‘simple mastectomy’’[All Fields] OR ‘‘mastectomy’’[All Fields] OR ‘‘mastectomy’’[MeSH Terms]))

*Search nr 2 (Results: 292)*

1. "Negative-Pressure Wound Therapy"[Mesh]
2. activac[tiab] OR "activ a c"[tiab] OR "avance solo"[tiab:~0] OR avelle[tiab] OR "foam suction"[tiab] OR "negative pressure"[tiab] OR "negative suction"[tiab] OR "negative wound pressure"[tiab] OR npt[tiab] OR npwt[tiab] OR nwpt[tiab] OR prevena[tiab] OR renasys[tiab] OR "suction dressing*"[tiab] OR tnp[tiab] OR vac[tiab] OR "v a c"[tiab] OR "vacuum assisted closure"[tiab:~1] OR "vacuum assisted dressing"[tiab:~1] OR "vacuum assisted dressings"[tiab:~1] OR "vacuum assisted therap*"[tiab] OR "vacuum dressing*"[tiab] OR "vacuum seal* drainage"[tiab] OR "vacuum therap*"[tiab] OR veraflo[tiab]
3. pico[tiab] OR "negative atmospheric"[tiab] OR "sub atmospheric"[tiab] OR subatmospheric[tiab]
4. wound[tiab] OR dressing*[tiab]
5. #3 AND #4
6. #1 OR #2 OR #5
7. "Mammaplasty"[Mesh] OR "Mastectomy"[Mesh]
8. "breast augmentation*"[tiab] OR "breast conserv*"[tiab] OR "breast enlarg*"[tiab] OR "breast implant*"[tiab] OR "breast lift*"[tiab] OR "breast prosthes*"[tiab] OR "breast reduction*"[tiab] OR "breast reconstruction*"[tiab] OR "breast sparing"[tiab] OR "chest femini*"[tiab] OR "chest masculini*"[tiab] OR lumpectom*[tiab] OR mammectom*[tiab] OR mammaplast*[tiab] OR mammoplast*[tiab] OR mastectom*[tiab] OR mastopex*[tiab] OR quadrantectom*[tiab] OR segmentectom*[tiab] OR postlumpectom*[tiab] OR postmammectom*[tiab] OR postmammaplast*[tiab] OR postmammoplast*[tiab] OR postmastectom*[tiab] OR postquadrantectom*[tiab] OR postsegmentectom*[tiab] OR "top surger*"[tiab]
9. #7 OR #8
10. "Breast"[Mesh] OR "Breast Neoplasms"[Mesh]
11. breast[tiab] OR breasts[tiab]
12. #10 OR #11
13. "Surgical Procedures, Operative"[Mesh] OR "surgery"[MeSH Subheading]
14. intraoperati*[tiab] OR oncoplast*[tiab] OR operati*[tiab] OR perioperati*[tiab] OR peroperati*[tiab] OR postoperati*[tiab] OR surg*[tiab]
15. #13 OR #14
16. #12 AND #15
17. #9 OR #16
18. #6 AND #17

**Embase**

*Results: 363*

1. 'vacuum assisted closure device'/de/mj OR 'vacuum assisted closure'/de/mj
2. (activac OR 'activ a c' OR 'avance solo' OR avelle OR 'foam suction' OR 'negative pressure' OR 'negative suction' OR 'negative wound pressure' OR npt OR npwt OR nwpt OR prevena OR renasys OR 'suction dressing*' OR tnp OR vac OR 'v a c' OR ('vacuum assisted' NEAR/1 (closure OR dressing$ OR therap*)) OR 'vacuum dressing*' OR 'vacuum seal* drainage' OR 'vacuum therap*' OR veraflo OR ((pico OR 'negative atmospheric' OR 'sub atmospheric' OR subatmospheric) NEAR/3 (wound OR dressing*))):ti,ab,kw
3. #1 OR #2
4. 'breast surgery'/exp/mj
5. ('breast augmentation*' OR 'breast conserv*' OR 'breast enlarg*' OR 'breast implant*' OR 'breast lift*' OR 'breast prosthes*' OR 'breast reduction*' OR 'breast reconstruction*' OR 'breast sparing' OR 'chest femini*' OR 'chest masculini*' OR lumpectom* OR mammectom* OR mammaplast* OR mammoplast* OR mastectom* OR mastopex* OR quadrantectom* OR segmentectom* OR postlumpectom* OR postmammectom* OR postmammaplast* OR postmammoplast* OR postmastectom* OR postquadrantectom* OR postsegmentectom* OR 'top surger*'):ti,ab,kw
6. #4 OR #5
7. 'breast'/de/mj OR 'breast cancer'/exp/mj
8. (breast$):ti,ab,kw
9. #7 OR #8
10. 'surgery'/exp/mj
11. (intraoperati* OR oncoplast* OR operati* OR perioperati* OR peroperati* OR postoperati* OR surg*):ti,ab,kw
12. #10 OR #11
13. #9 AND #11
14. #6 OR #13
15. #3 AND #14

**Cochrane library**

*Results: 95*

1. "Negative-Pressure Wound Therapy"[Mesh]
2. (activac OR "activ a c" OR "avance solo" OR avelle OR "foam suction" OR "negative pressure" OR "negative suction" OR "negative wound pressure" OR npt OR npwt OR nwpt OR prevena OR renasys OR (suction NEXT dressing*) OR tnp OR vac OR "v a c" OR ("vacuum assisted" NEAR/1 (closure OR dressing? OR therap*)) OR (vacuum NEXT dressing*) OR (vacuum NEXT seal* NEXT drainage) OR (vacuum NEXT therap*) OR veraflo OR ((pico OR "negative atmospheric" OR "sub atmospheric" OR subatmospheric) NEAR/3 (wound OR dressing*))):ti,ab,kw
3. 3 #1 OR #2
4. "Mammaplasty"[Mesh] OR "Mastectomy"[Mesh]
5. ((breast NEXT (augmentation* OR conserv* OR enlarg* OR implant* OR lift* OR prosthes* OR reduction* OR reconstruction*)) OR "breast sparing" OR (chest NEXT (femini* OR masculini*)) OR lumpectom* OR mammectom* OR mammaplast* OR mammoplast* OR mastectom* OR mastopex* OR quadrantectom* OR segmentectom* OR postlumpectom* OR postmammectom* OR postmammaplast* OR postmammoplast* OR postmastectom* OR postquadrantectom* OR postsegmentectom* OR (top NEXT surger*)):ti,ab,kw
6. #4 OR #5
7. "Breast"[Mesh] OR "Breast Neoplasms"[Mesh]
8. (breast?):ti,ab,kw
9. #7 OR #8
10. "Surgical Procedures, Operative"[Mesh] OR "surgery"[MeSH Subheading]
11. (intraoperati* OR oncoplast* OR operati* OR perioperati* OR peroperati* OR postoperati* OR surg*):ti,ab,kw
12. #10 OR #11
13. #9 AND #11
14. #6 OR #13
15. #3 AND #14

**Figure S1.**

**Contour- enhanced funnel plot assessing publication bias in studies reporting wound dehiscence**

**Figure S2.**

**Contour- enhanced funnel plot assessing publication bias in studies reporting surgical site infection**

**Figure S3.**

**Forest plot illustrating the effect of incisional Negative Pressure Wound Therapy (iNPWT) on prevention of seroma**

**Figure S4.**

**Contour- enhanced funnel plot assessing publication bias in studies reporting seroma**

**Figure S5.**

**Forest plot illustrating the effect of incisional Negative Pressure Wound Therapy (iNPWT) on prevention of skin necrosis**

**Figure S6.**

**Contour- enhanced funnel plot assessing publication bias in studies reporting skin necrosis**

**Figure S7.**

**Forest plot illustrating the effect of incisional Negative Pressure Wound Therapy (iNPWT) on prevention of nipple-areolar complex (NAC) necrosis**

**Figure S8.**

**Contour- enhanced funnel plot assessing publication bias in studies reporting nipple-areolar complex (NAC) necrosis**

**Figure S9.**

**Forest plot illustrating the effect of incisional Negative Pressure Wound Therapy (iNPWT) on prevention of hematoma**

**Figure S10.**

**Contour- enhanced funnel plot assessing publication bias in studies reporting hematoma**

**Table S1. Intervention-related characteristics of included studies: incisional Negative Pressure Wound Therapy (iNPWT) device, applied pressure (mmHg), dressing duration, follow-up period, and industry funding**

| **Study** | **iNPWT Device** | **mm Hg** | **Min. Days** | **Follow-Up (Days)** | **Industry Funding** |
| --- | --- | --- | --- | --- | --- |
| Abu El Hawa AA. Et al^32^ | Prevena | -120 | 7 | 7 | No |
| Akhter HM. et al^33^ | Prevena | Not Reported | Not Reported | Not Reported | No |
| Al-Ishaq Z. et al^34^ | PICO | -80 | 7 | Not Reported | Not Reported |
| Alameddine K. et al^35^ | Not Reported | Not Reported | Not Reported | Not Reported | Not Reported |
| Alameddine KO. et al^36^ | Prevena | -125 | 7 | 803 | No |
| Casella D. et al^37^ | Not Reported | -80 | Not Reported | 60 | No |
| De Rooij L. et al^38^ | Avelle | -80 | 7 | 90 | Yes |
| Diaz R. et al^39^ | PICO | -80 | 7 | Not Reported | Not Reported |
| Esen E. et al^40^ | Prevena | -90 increased to -120 | 4 | Not Reported | No |
| Fernandes U. et al^41^ | Not Reported | Not Reported | Not Reported | Not Reported | Not Reported |
| Ferrando PM. et al^59^ | Prevena | -125 | 7 | 365 | No |
| Fogacci T. et al^42^ | PICO | -80 | Not Reported | Not Reported | Not Reported |
| Gabriel A. et al^43^ | Prevena | -125 | 7 | 90 | Yes |
| Galiano R. et al^44^ | PICO | -80 | 7 | 21 | Yes |
| Holt R. et al^45^ | PICO | -80 | 6 | 12 | Not Reported |
| Irwin G. W. et al^46^ | PICO | -80 | Not Reported | Not Reported | Not Reported |
| Johnson O.N. 3rd et al^47^ | Prevena | -125 | 7 | 600 | No |
| Kim DY. et al^48^ | Not Reported | -125 | 3 | Not Reported | Not Reported |
| Lane A. et al^49^ | Not Reported | Not Reported | Not Reported | Not Reported | Not Reported |
| Lauritzen E. et al^50^ | Prevena | -125 | 7 | 12 | No |
| Nip L. et al^51^ | Not Reported | Not Reported | Not Reported | 30 | No |
| Ockerman KM. et al^52^ | Prevena | -125 | 7 | 90 | Yes |
| Pellino G. et al^53^ | PICO | -80 | 7 | 90 | No |
| Pieri A. et al^54^ | Prevena | Not Reported | Not Reported | Not Reported | Not Reported |
| Pieszko K. et al^55^ | Avelle | -80 | 7 | 365 | Yes |
| Ryu JY. et al^56^ | PICO | -80 | 7 | Not Reported | Not Reported |
| Timmermans FW. et al^15^ | PICO | -80 | 7 | 365 | No |
| Tormey S. et al^57^ | Not Reported | Not Reported | Not Reported | Not Reported | Not Reported |
| Wareham CM. et al^58^ | Prevena | -125 | Not Reported | 730 | Yes |

**Table S2. Mixed-effects meta-regression model: predictors of incisional Negative Pressure Wound Therapy (NPWT) effect on different outcomes**

| **Outcome** | **Variable** | **Coefficient (β)** | **Std. Err.** | **95% Confidence Interval** | **P-value** |
| --- | --- | --- | --- | --- | --- |
| **Wound Dehiscence** | Intercept | -9.774 | 5.208 | -19.981 to 0.433 | 0.061 |
|  | Smoking | -4.543 | 16.806 | -37.482 to 28.396 | 0.787 |
|  | Age | -0.007 | 0.039 | -0.084 to 0.070 | 0.860 |
|  | BMI | 0.265 | 0.207 | -0.141 to 0.672 | 0.200 |
| **Surgical Site Infection** | Intercept | 3.262 | 5.074 | -6.684 to 13.208 | 0.520 |
|  | Smoking | 2.755 | 6.708 | -10.392 to 15.902 | 0.681 |
|  | Age | 0.042 | 0.041 | -0.038 to 0.121 | 0.306 |
|  | BMI | -0.302 | 0.174 | -0.643 to 0.038 | 0.082 |
| **Seroma** | Intercept | -2.072 | 1.072 | -4.172 to 0.029 | 0.053 |
|  | NPWT device: PICO | 0.131 | 0.115 | -0.094 to 0.356 | 0.253 |
|  | NPWT device: Avelle | 0.838 | 0.366 | 0.121 to 1.554 | 0.022 |
|  | Mastectomy | 0.130 | 0.094 | -0.053 to 0.314 | 0.164 |
|  | Smoking | 0.329 | 0.997 | -1.625 to 2.284 | 0.741 |
|  | Age | -0.001 | 0.004 | -0.009 to 0.006 | 0.664 |
|  | BMI | 0.007 | 0.039 | -0.006 to 0.147 | 0.071 |
| **Skin Necrosis** | Intercept | -2.066 | 3.784 | -9.483 to 5.351 | 0.585 |
|  | Smoking | 6.879 | 18.091 | -28.578 to 42.338 | 0.704 |
|  | Age | -0.188 | 0.054 | -0.818 to -0.015 | 0.039 |
|  | BMI | -0.169 | 0.103 | -0.249 to -0.080 | 0.042 |
| **Hematoma** | Intercept | -1.487 | 5.440 | -12.149 to 9.175 | 0.785 |
|  | Smoking | -2.774 | 9.357 | -21.113 to 15.565 | 0.767 |
|  | Age | -0.021 | 0.054 | -0.127 to 0.085 | 0.697 |
|  | BMI | -0.054 | 0.178 | -0.404 to 0.296 | 0.762 |

**Table S3. Risk of bias assessment using the Cochrane Risk of Bias-2 (RoB2) tool across included randomized controlled trials
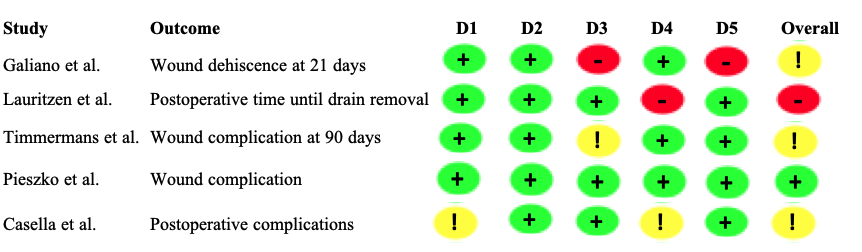
**

**Abbreviations:**

Low risk of bias

Some concerns

High risk of bias

**D1**: Bias arising from the randomization process

**D2**: Bias due to deviations from intended interventions

**D3**: Bias due to missing outcome data

**D4**: Bias in measurement of the outcome

**D5**: Bias in selection of the reported result

**Table S4. Risk of bias assessment using ROBINS-I tool across included non-randomized studies**

| **Study** | **D1** | **D2** | **D3** | **D4** | **D5** | **D6** | **D7** | **Overall Risk** |
| --- | --- | --- | --- | --- | --- | --- | --- | --- |
| Abu El Hawa AA. et al.^32^ | 🟡 | 🟡 | 🟢 | 🟢 | 🟢 | 🟡 | 🟢 | 🟡 |
| Akhter HM et al.^33^ | 🟠 | 🟡 | 🟢 | 🟢 | 🟢 | 🟡 | 🟢 | 🟠 |
| Al-Ishaq Z. et al.^34^ | 🟠 | 🔴 | 🟢 | 🟢 | 🟢 | 🟡 | 🟠 | 🔴 |
| Alameddine K. et al.^35^ | 🟢/🟡 | 🟡 | 🟢 | 🟢 | 🟢 | 🟡 | 🟢 | 🟡 |
| Alameddine KO et al.^36^ | 🔴 | 🔴 | 🟢 | 🟢 | 🟡 | 🟡 | 🟠 | 🔴 |
| De Rooij L. et al.^14^ | 🟢/🟡 | 🟡 | 🟢 | 🟢 | 🟢 | 🟡 | 🟢 | 🟡 |
| Diaz R. et al.^39^ | 🔴 | 🔴 | 🟢 | 🟢 | 🟢 | 🟡 | 🟠 | 🔴 |
| Esen E. et al.^40^ | 🔴 | 🔴 | 🟢 | 🟢 | 🟡 | 🟡 | 🟠 | 🔴 |
| Fernandes U. et al.^41^ | 🔴 | 🔴 | 🟢 | 🟢 | 🟡 | 🟡 | 🟠 | 🔴 |
| Fogacci T. et al.^42^ | 🔴 | 🔴 | 🟢 | 🟢 | 🟢 | 🟡 | 🟠 | 🔴 |
| Gabriel A. et al.^43^ | 🟡 | 🔴 | 🟢 | 🟢 | 🟢 | 🟡 | 🟢/🟡 | 🟠 |
| Holt R. et al.^45^ | 🟠 | 🟡 | 🟢 | 🟢 | 🟢 | 🟡 | 🟠 | 🟠 |
| Irwin G. W. et al.^46^ | 🟡 | 🟢 | 🟢 | 🟢/🟡 | 🟢 | 🟡 | 🟢 | 🟡 |
| Johnson O.N. et al.^47^ | 🟡 | 🟡 | 🟢 | 🟢 | 🟢 | 🟡 | 🟢/🟡 | 🟡 |
| Kim DY et al.^48^ | 🟡 | 🟡 | 🟢 | 🟢 | 🟢 | 🟡 | 🟢/🟡 | 🟡 |
| Lane A. et al.^49^ | 🔴 | 🟠 | 🟢 | 🟢 | 🟢 | 🟡 | 🟠 | 🔴 |
| Nip L. et al.^51^ | 🔴 | 🔴 | 🟢 | 🟢/🟡 | 🟡 | 🟡 | 🔴 | 🔴 |
| Ockerman KM. et al.^52^ | 🟠 | 🟡 | 🟢 | 🟢/🟡 | 🟡 | 🟡 | 🟢/🟡 | 🟠 |
| Pellino G. et al.^53^ | 🔴 | 🟡 | 🟢 | 🟢 | 🟢 | 🟡 | 🟠 | 🔴 |
| Pieri A. et al.^54^ | 🔴 | 🟠 | 🟢 | 🟢/🟡 | 🟢 | 🟡 | 🟢/🟡 | 🔴 |
| Ryu JY. et al.^56^ | 🟡 | 🟡 | 🟢 | 🟢 | 🟢 | 🟡 | 🟢/🟡 | 🟡 |
| Tormey S. et al.^57^ | 🔴 | 🟡 | 🟢 | 🟠 | 🟢 | 🟡 | 🟠 | 🔴 |
| Wareham CM. et al.^58^ | 🟠 | 🔴 | 🟢 | 🟢/🟡 | 🟡 | 🟠 | 🟢/🟡 | 🔴 |
| Ferrando PM. et al.^59^ | 🟠 | 🟠 | 🟢 | 🟢 | 🟢 | 🟡 | 🟠 | 🟠 |

**Abbreviations:**

| 🟢 | Low |
| --- | --- |
| 🟢/🟡 | Low–Moderate |
| 🟡 | Moderate |
| 🟠 | Moderate–Serious |
| 🔴 | Serious |

| **D1**: | Bias due to confounding |
| --- | --- |
| **D2**: | Bias in selection of participants into the study |
| **D3**: | Bias in classification of interventions |
| **D4**: | Bias due to deviations from intended interventions |
| **D5**: | Bias due to missing data |
| **D6**: | Bias in measurement of outcomes |
| **D7**: | Bias in selection of the reported result |

**Table S5. Assessment of certainty of evidence using the GRADE (grading of recommendations, assessment, development and evaluations) framework**

| **Outcome** | **Study designs** | **Risk of bias** | **Inconsistency** | **Imprecision** | **Publication bias** | **Overall certainty of evidence** | **Downgrading rationale** |
| --- | --- | --- | --- | --- | --- | --- | --- |
| **Wound dehiscence** | RCT + Non-RCT | Not serious (RCT) / Serious (Non-RCT) | Serious (I² = 81.6%) | Not serious | Possible (Egger p = 0.112) | ⬤⬤⬤◯ Moderate | Downgraded for inconsistency and publication bias |
| **Surgical site infection** | RCT + Non-RCT | Not serious (RCT) / Serious (Non-RCT) | Serious (I² = 84.1%) | Not serious | Detected (Egger p = 0.006) | ⬤⬤◯◯ Low | Downgraded for inconsistency and publication bias |
| **Seroma** | Non-RCT | Serious | Serious (I² = 96.7%) | Serious (CI crosses null) | Detected (Egger p = 0.003) | ⬤◯◯◯ Very low | Downgraded for risk of bias, inconsistency, and imprecision |
| **Skin necrosis** | Non-RCT | Serious | Serious (I² = 87.8%) | Serious | Not detected (Egger p = 0.25) | ⬤⬤◯◯ Low | Downgraded for risk of bias and inconsistency |
| **Nipple-areolar complex necrosis** | Non-RCT | Serious | Serious (I² = 91.1%) | Serious (CI crosses null) | Not detected (Egger p = 0.089) | ⬤◯◯◯ Very low | Downgraded for risk of bias, inconsistency, and imprecision |
| **Hematoma** | Non-RCT | Serious | Moderate (I² = 59.5%) | Serious (CI crosses null) | Detected (Egger p = 0.009) | ⬤⬤◯◯  Low | Downgraded for bias and publication bias |

**Table S6. Sensitivity analysis for net Cost per SSI Prevented (€) for different scenarios.**

| **Scenario** | **Absolute Risk Difference (ARD)** | **Number Needed to Treat** | **SSI Cost (€)** | **Incremental Treatment Cost (€)** | **Net Cost per SSI Prevented (€)** |
| --- | --- | --- | --- | --- | --- |
| Low ARD & Low SSI Cost | 0.020 | 50 | € 3481 | € 211 | (50 × 211) - 3481 = 10550 - 3481 = **7069** |
| Low ARD & Median SSI Cost | 0.020 | 50 | € 10005 | € 211 | (50 × 211) - 10005 = 10550 - 10005 = **545** |
| Median ARD & Low SSI Cost | 0.048 | 21 | € 3481 | € 211 | (21 × 211) - 3481 = 4431 - 3481 = **950** |
| Median ARD & Median SSI Cost | 0.048 | 21 | € 10005 | € 211 | (21 × 211) - 10005 = 4431 - 10005 = **-5574** |

**References**

1. Cagney D, Simmons L, O'Leary DP, Corrigan M, Kelly L, O'Sullivan MJ, Liew A, Redmond HP. The Efficacy of Prophylactic Negative Pressure Wound Therapy for Closed Incisions in Breast Surgery: A Systematic Review and Meta-Analysis. *World J Surg* 2020;**44**(5): 1526-1537.

2. Thalji SZ, Cortina CS, Guo MS, Kong AL. Postoperative Complications from Breast and Axillary Surgery. *Surgical Clinics of North America* 2023;**103**(1): 121-139.

3. Adwall L, Fredriksson I, Hultin H, Mani M, Norlén O. Postoperative complications after breast cancer surgery and effect on recurrence and survival: population-based cohort study. *BJS Open* 2024;**8**(6): zrae137.

4. Gupta S, King WD, Korzeniowski M, Wallace DL, Mackillop WJ. The Effect of Waiting Times for Postoperative Radiotherapy on Outcomes for Women Receiving Partial Mastectomy for Breast Cancer: a Systematic Review and Meta-Analysis. *Clin Oncol (R Coll Radiol)* 2016;**28**(12): 739-749.

5. Dinapoli L, Colloca G, Di Capua B, Valentini V. Psychological Aspects to Consider in Breast Cancer Diagnosis and Treatment. *Curr Oncol Rep* 2021;**23**(3): 38.

6. Chavez-MacGregor M, Clarke CA, Lichtensztajn DY, Giordano SH. Delayed Initiation of Adjuvant Chemotherapy Among Patients With Breast Cancer. *JAMA Oncol* 2016;**2**(3): 322-329.

7. Olsen MA, Chu-Ongsakul S, Brandt KE, Dietz JR, Mayfield J, Fraser VJ. Hospital-associated costs due to surgical site infection after breast surgery. *Arch Surg* 2008;**143**(1): 53-60; discussion 61.

8. Groenen H, Jalalzadeh H, Buis DR, Dreissen YEM, Goosen JHM, Griekspoor M, Harmsen WJ, FFA IJ, van der Laan MJ, Schaad RR, Segers P, van der Zwet WC, de Jonge SW, Orsini RG, Eskes AM, Wolfhagen N, Boermeester MA. Incisional negative pressure wound therapy for the prevention of surgical site infection: an up-to-date meta-analysis and trial sequential analysis. *EClinicalMedicine* 2023;**62**: 102105.

9. Vikatmaa P, Juutilainen V, Kuukasjärvi P, Malmivaara A. Negative pressure wound therapy: a systematic review on effectiveness and safety. *Eur J Vasc Endovasc Surg* 2008;**36**(4): 438-448.

10. Agarwal P, Kukrele R, Sharma D. Vacuum assisted closure (VAC)/negative pressure wound therapy (NPWT) for difficult wounds: A review. *J Clin Orthop Trauma* 2019;**10**(5): 845-848.

11. Orgill DP, Manders EK, Sumpio BE, Lee RC, Attinger CE, Gurtner GC, Ehrlich HP. The mechanisms of action of vacuum assisted closure: more to learn. *Surgery* 2009;**146**(1): 40-51.

12. Kairinos N, Voogd AM, Botha PH, Kotze T, Kahn D, Hudson DA, Solomons M. Negative-pressure wound therapy II: negative-pressure wound therapy and increased perfusion. Just an illusion? *Plast Reconstr Surg* 2009;**123**(2): 601-612.

13. Molska M, Wojciech M, Murawa D. Comparison of Single-Use Negative-Pressure Wound Therapy (sNPWT) and Standard Dressings Applied to the Same Patient During Bilateral Tissue Expander-to-Implant Exchanges. *Cancers (Basel)* 2024;**17**(1).

14. De Rooij L, Bosmans J, van Kuijk SMJ, Vissers YLJ, Beets GL, van Bastelaar J. A systematic review of seroma formation following drain-free mastectomy. *Eur J Surg Oncol* 2021;**47**(4): 757-763.

15. Timmermans FW, Mokken SE, Smit JM, Zwanenburg PR, van Hout N, Bouman MB, Middelkoop E, Mullender MG. Within-patient randomized clinical trial comparing incisional negative-pressure wound therapy with suction drains in gender-affirming mastectomies. *Br J Surg* 2021;**108**(8): 925-933.

16. Liew AN, Lim KY, Khoo JF. Closed Incision Negative Pressure Therapy vs Standard of Care Dressing in Breast Surgery: A Systematic Review. *Cureus* 2022;**14**(4): e24499.

17. Page MJ, McKenzie JE, Bossuyt PM, Boutron I, Hoffmann TC, Mulrow CD, Shamseer L, Tetzlaff JM, Akl EA, Brennan SE, Chou R, Glanville J, Grimshaw JM, Hróbjartsson A, Lalu MM, Li T, Loder EW, Mayo-Wilson E, McDonald S, McGuinness LA, Stewart LA, Thomas J, Tricco AC, Welch VA, Whiting P, Moher D. The PRISMA 2020 statement: an updated guideline for reporting systematic reviews. *BMJ* 2021;**372**: n71.

18. McKenzie JB, SE; Ryan, RE; Thomson, HJ; Johnston, RV; Thomas, J. Chapter 3: Defining the criteria for including studies and how they will be grouped for the synthesis. In: *Cochrane Handbook for Systematic Reviews of Interventions version 65*, Higgins JT, J; Chandler, J; Cumpston, M; Li, T; Page, MJ; Welch, VA (ed). Cochrane, 2024.

19. Ouzzani M, Hammady H, Fedorowicz Z, Elmagarmid A. Rayyan-a web and mobile app for systematic reviews. *Syst Rev* 2016;**5**(1): 210.

20. Weber F, Knapp G, Glass Ä, Kundt G, Ickstadt K. Interval estimation of the overall treatment effect in random-effects meta-analyses: Recommendations from a simulation study comparing frequentist, Bayesian, and bootstrap methods. *Res Synth Methods* 2021;**12**(3): 291-315.

21. Borenstein M, Hedges LV, Higgins JP, Rothstein HR. A basic introduction to fixed-effect and random-effects models for meta-analysis. *Res Synth Methods* 2010;**1**(2): 97-111.

22. IntHout J, Ioannidis JPA, Rovers MM, Goeman JJ. Plea for routinely presenting prediction intervals in meta-analysis. *BMJ Open* 2016;**6**(7): e010247.

23. Murad MH, Wang Z, Zhu Y, Saadi S, Chu H, Lin L. Methods for deriving risk difference (absolute risk reduction) from a meta-analysis. *BMJ* 2023;**381**: e073141.

24. Nyaga VN, Arbyn M, Aerts M. Metaprop: a Stata command to perform meta-analysis of binomial data. *Archives of Public Health* 2014;**72**(1): 39.

25. Borenstein M, Higgins JP, Hedges LV, Rothstein HR. Basics of meta-analysis: I(2) is not an absolute measure of heterogeneity. *Res Synth Methods* 2017;**8**(1): 5-18.

26. Hu B, Shao J, Palta M. PSEUDO-R <sup>2</sup> IN LOGISTIC REGRESSION MODEL. *Statistica Sinica* 2006;**16**(3): 847-860.

27. Sterne JAC, Savović J, Page MJ, Elbers RG, Blencowe NS, Boutron I, Cates CJ, Cheng HY, Corbett MS, Eldridge SM, Emberson JR, Hernán MA, Hopewell S, Hróbjartsson A, Junqueira DR, Jüni P, Kirkham JJ, Lasserson T, Li T, McAleenan A, Reeves BC, Shepperd S, Shrier I, Stewart LA, Tilling K, White IR, Whiting PF, Higgins JPT. RoB 2: a revised tool for assessing risk of bias in randomised trials. *Bmj* 2019;**366**: l4898.

28. Sterne JA, Hernán MA, Reeves BC, Savović J, Berkman ND, Viswanathan M, Henry D, Altman DG, Ansari MT, Boutron I, Carpenter JR, Chan AW, Churchill R, Deeks JJ, Hróbjartsson A, Kirkham J, Jüni P, Loke YK, Pigott TD, Ramsay CR, Regidor D, Rothstein HR, Sandhu L, Santaguida PL, Schünemann HJ, Shea B, Shrier I, Tugwell P, Turner L, Valentine JC, Waddington H, Waters E, Wells GA, Whiting PF, Higgins JP. ROBINS-I: a tool for assessing risk of bias in non-randomised studies of interventions. *Bmj* 2016;**355**: i4919.

29. Guyatt GH, Oxman AD, Vist GE, Kunz R, Falck-Ytter Y, Alonso-Coello P, Schünemann HJ. GRADE: an emerging consensus on rating quality of evidence and strength of recommendations. *Bmj* 2008;**336**(7650): 924-926.

30. Gupta N, Verma R, Dhiman RK, Rajsekhar K, Prinja S. Cost-Effectiveness Analysis and Decision Modelling: A Tutorial for Clinicians. *J Clin Exp Hepatol* 2020;**10**(2): 177-184.

31. Nilsson M, Hughes J, Styche T, Knudsen JT. Improvement in community wound management: why simple changes in clinical practice matter. *British Journal of Healthcare Management* 2024;**30**(5): 1-12.

32. Abu El Hawa AA, Dekker PK, Mizher R, Orra S, Fan KL, Del Corral G. Utility of Negative Pressure Wound Therapy: Raising the Bar in Chest Masculinization Surgery. *Plast Reconstr Surg Glob Open* 2022;**10**(2): e4096.

33. Akhter HM, Macdonald C, McCarthy P, Huang Y, Meyer BR, Shostrum VK, Cromer KJ, Johnson PJ, Wong SL, Hon HH. Outcomes of Negative Pressure Wound Therapy on Immediate Breast Reconstruction after Mastectomy. *Plast Reconstr Surg Glob Open* 2023;**11**(8): e5130.

34. Al-Ishaq Z, Rahman E, Salem F, Taj S, Mula-Hussain L, Mylvaganam S, Vidya R, Matey P, Sircar T. Is Using Closed Incision Negative Pressure Therapy in Reconstructive and Oncoplastic Breast Surgery Helpful in Reducing Skin Necrosis? *Cureus* 2023;**15**(4): e38167.

35. Alameddine K, Mohamed O, Chen A, Harless C. Risk of Complications After Breast Surgery in Radiated Patients Using the Closed Incision Negative Pressure Therapy Dressings Compared to Standard-of-Care Dressings. *Aesthet Surg J Open Forum* 2023;**5**(Suppl 1).

36. Alameddine KO, Salinas CA, Yan M, Martinez-Jorge J, Vijayasekaran A, Tran NV, Harless CA. Efficacy of Closed-Incision Negative Pressure Wound Therapy in Reducing Postoperative Complications in Breast Reconstruction After Radiotherapy: A Propensity Score Analysis. *Aesthet Surg J Open Forum* 2024;**6**: ojae073.

37. Casella D, Fusario D, Pesce AL, Marcasciano M, Lo Torto F, Luridiana G, De Luca A, Cuomo R, Ribuffo D. Portable Negative Pressure Wound Dressing in Oncoplastic Conservative Surgery for Breast Cancer: A Valid Ally. *Medicina (Kaunas)* 2023;**59**(10).

38. De Rooij L, van Kuijk SMJ, van Haaren ERM, Janssen A, Vissers YLJ, Beets GL, van Bastelaar J. Negative pressure wound therapy does not decrease postoperative wound complications in patients undergoing mastectomy and flap fixation. *Sci Rep* 2021;**11**(1): 9620.

39. Diaz R, Cuniolo L, Marassi G, Murelli F, Depaoli F, Gipponi M, Cornacchia C, Margarino C, Boccardo C, Franchelli S, Pesce M, Baldelli I, Picardi S, Piccioli M, De Cian F, Fregatti P. Negative Pressure Wound Therapy (NPWT) in breast surgery: our preliminary experience. *European Journal of Surgical Oncology* 2024.

40. Esen E, Morkavuk SB, Turan M, Akyuz S, Guler S, Akgul GG, Bahcecioglu IB, Gulcelik MA, Yilmaz KB. The use of incisional negative pressure wound therapy on high-risk breast cancer mastectomy patients. *Asian J Surg* 2024.

41. Fernandes U, Marçal A, Pereira R, Guidi G, Martins D, Vieira B, Leal C, Marques R, Ferreira C, Silva S, Melo A, Avelar P, Esteves A, Pinto-de-Sousa J. The Impact of Closed Incision Negative Pressure Therapy on Postoperative Oncologic Breast Surgery Outcomes. *European Journal of Surgical Oncology* 2021;**47**(2): e45.

42. Fogacci T, Cattin F, Samorani D. The negative pressure therapy with PICO as a prevention of surgical site infection in high risk patients undergoing breast surgery. *Annals of Oncology* 2019;**30**: iii42.

43. Gabriel A, Sigalove S, Sigalove N, Storm-Dickerson T, Rice J, Maxwell P, Griffin L. The Impact of Closed Incision Negative Pressure Therapy on Postoperative Breast Reconstruction Outcomes. *Plast Reconstr Surg Glob Open* 2018;**6**(8): e1880.

44. Galiano RD, Hudson D, Shin J, van der Hulst R, Tanaydin V, Djohan R, Duteille F, Cockwill J, Megginson S, Huddleston E. Incisional Negative Pressure Wound Therapy for Prevention of Wound Healing Complications Following Reduction Mammaplasty. *Plast Reconstr Surg Glob Open* 2018;**6**(1): e1560.

45. Holt R, Shotton R, Murphy J. P130. Negative pressure wound therapy (NPWT) on complex closed breast incisions promotes wound healing. *European Journal of Surgical Oncology (EJSO)* 2015;**41**: S63.

46. Irwin GW, Boundouki G, Fakim B, Johnson R, Highton L, Myers D, Searle R, Murphy JA. Negative Pressure Wound Therapy Reduces Wound Breakdown and Implant Loss in Prepectoral Breast Reconstruction. *Plast Reconstr Surg Glob Open* 2020;**8**(2): e2667.

47. Johnson ON, 3rd, Reitz CL, Thai K. Closed Incisional Negative Pressure Therapy Significantly Reduces Early Wound Dehiscence after Reduction Mammaplasty. *Plast Reconstr Surg Glob Open* 2021;**9**(3): e3496.

48. Kim DY, Park SJ, Bang SI, Mun GH, Pyon JK. Does the Use of Incisional Negative-Pressure Wound Therapy Prevent Mastectomy Flap Necrosis in Immediate Expander-Based Breast Reconstruction? *Plast Reconstr Surg* 2016;**138**(3): 558-566.

49. Lane A, Flor RJ, Reitz C, Thai K, Johnson ON. Incisional Negative Pressure Wound Therapy: A Novel Technique to Reduce Early Wound Dehiscence Morbidity of High-Risk Breast Incisions. *Journal of the American College of Surgeons* 2018;**227**(4, Supplement 1): S184-S185.

50. Lauritzen E, Kiilerich CH, Bredgaard R, Tvedskov T, Damsgaard TE. Incisional negative pressure wound therapy (PrevenaTM) vs. conventional post-operative dressing after immediate breast reconstruction: a randomized controlled clinical trial. *European Journal of Plastic Surgery* 2024;**47**(1): 79.

51. Nip L, Fatayer H, Rusius V, Bramley M. P091: Surgical Site Infection (SSI) and seroma rates in oncoplastic breast patients using negative pressure wound dressings. *European Journal of Surgical Oncology* 2020;**46**(6): e34-e35.

52. Ockerman KM, Bryan J, Wiesemann G, Neal D, Marji FP, Heath F, Kanchwala S, Oladeru O, Spiguel L, Sorice-Virk S. Closed Incision Negative Pressure Therapy in Oncoplastic Surgery Prevents Delays to Adjuvant Therapy. *Plast Reconstr Surg Glob Open* 2023;**11**(5): e5028.

53. Pellino G, Sciaudone G, Candilio G, De Fatico GS, Landino I, Della Corte A, Guerniero R, Benevento R, Santoriello A, Campitiello F, Selvaggi F, Canonico S. Preventive NPWT over closed incisions in general surgery: does age matter? *Int J Surg* 2014;**12 Suppl 2**: S64-s68.

54. Pieri A, Eves A, Kay K, Irving J, Robert T, Cain H, Kalra L, Critchley A. Managing No-Drain Mastectomy with Closed Incision Negative Pressure Wound Therapy using Full-Coverage Foam Dressings. *European Journal of Surgical Oncology* 2023;**49**(2): e94.

55. Pieszko K, Pieszko K, Wichtowski M, Cieśla S, Ławnicka A, Jamont R, Boyd JB, Murawa D. A Randomized Study Comparing Closed-Incision Negative-Pressure Wound Therapy with Standard Care in Immediate Breast Reconstruction. *Plast Reconstr Surg* 2023;**151**(6): 1123-1133.

56. Ryu JY, Lee JH, Kim JS, Lee JS, Lee JW, Choi KY, Chung HY, Cho BC, Yang JD. Usefulness of Incisional Negative Pressure Wound Therapy for Decreasing Wound Complication Rates and Seroma Formation Following Prepectoral Breast Reconstruction. *Aesthetic Plast Surg* 2022;**46**(2): 633-641.

57. Tormey S, Mathers H, Sloan S, Styche T, Whittall C. P126. Impact of single use negative pressure wound therapy on surgical incisions following breast surgery: a multi-centre evaluation. *European Journal of Surgical Oncology* 2019;**45**: 917.

58. Wareham CM, Karamchandani MM, Ku GC, Gaffney K, Sekigami Y, Persing SM, Homsy C, Nardello S, Chatterjee A. Closed Incision Negative Pressure Therapy in Oncoplastic Breast Surgery: A Comparison of Outcomes. *Plast Reconstr Surg Glob Open* 2023;**11**(4): e4936.

59. Ferrando PM, Ala A, Bussone R, Bergamasco L, Actis Perinetti F, Malan F. Closed Incision Negative Pressure Therapy in Oncological Breast Surgery: Comparison with Standard Care Dressings. *Plast Reconstr Surg Glob Open* 2018;**6**(6): e1732.
